# Evaluation of red-complex bacteria loads in complete denture patients: a pilot study

**DOI:** 10.1038/s41405-023-00133-z

**Published:** 2023-02-18

**Authors:** Enis Veseli, Gloria Staka, Marcos Roberto Tovani-Palone

**Affiliations:** 1 Department of Prosthodontics, Dental School, Faculty of Medicine, University of Pristina, Pristina, Kosovo; 2 University Dentistry Clinical Center of Kosovo, Pristina, Kosovo; 3 Department of Research Analytics, Saveetha Dental College and Hospitals, Saveetha Institute of Medical and Technical Sciences, Chennai, India

**Keywords:** Health care, Oral anatomy

## Abstract

**Objective:**

This pilot study aimed to evaluate red-complex bacteria (RCB) loads in edentulous patients, before and after dentures’ insertion.

**Materials and methods:**

Thirty patients were included in the study. Deoxyribonucleic acid (DNA) isolated from bacterial samples were obtained from the dorsum of the tongue before and 3 months after complete dentures (CDs) insertion in order to identify the presence of RCB (*Tannerella forsythia, Porphyromonas gingivalis, and Treponema denticola)* and quantify their loads, using real-time polymerase chain reaction (RT-PCR). Bacterial loads were represented as “Lg (genome equivalents/sample)” and the data classified according to the “ParodontoScreen” test.

**Results:**

Significant changes in bacterial loads were observed before and 3 months after the CDs insertion for: *P. gingivalis* (0.40 ± 0.90 vs 1.29 ± 1.64, *p* = 0.0007), *T. forsythia* (0.36 ±0.94 vs 0.87 ± 1.45, *p* = 0.005), and *T. denticola* (0.11 ± 0.41 vs 0.33 ± 0.75, *p* = 0.03). Before the CDs insertion, all patients had a normal bacterial prevalence range (100%) for all analyzed bacteria. Three months after the insertion, 2 (6.7%) of them had a moderate bacterial prevalence range for *P. gingivalis*, while 28 (93.3%) had a normal bacterial prevalence range.

**Conclusion:**

The use of CDs has a significant impact on increasing RCB loads in edentulous patients.

## Introduction

Oral flora in edentulous patients is becoming a topic of great interest in dentistry, since dental implants have been increasingly used for prosthodontic rehabilitation. Although dental implant prosthesis is considered an excellent treatment option for the rehabilitation of edentulous patients, its failure can also occur, especially due to periimplantitis [1, 2]. Results from different studies have shown that the microbial component associated with dental implants failure involves periodontal pathogens that cause inflammation of peri-implant tissues, including red-complex bacteria (RCB)—*Tannerella forsythia, Porphyromonas gingivalis, and Treponema denticola* [3]. Therefore, a rigorous assessment of periodontal pathogens prior to rehabilitation of edentulous patients should be essential to reduce the chances of failure in the medium and long term.

Initially, it was believed that a full-mouth extraction would lead to the elimination of these pathogens [4]. However, this hypothesis has been overturned. With the advancement of molecular genetic techniques, researchers have found that periodontal pathogens are still present in edentulous mouths [5–7]. Recent research also suggests that some of these bacteria may be involved in the development of various systemic diseases, which can be high risk mainly for the elderly [8].

If, on the one hand, the interest of patients in dental implant prostheses has substantially increased nowadays, the demand for complete dentures (CDs) remains relatively high, either for financial reasons or for the general condition of the patient [9]. With an increasing burden of edentulous elderly people worldwide, it is expected that there will be an increase in patients seeking treatment with CDs [10].

It is worth noting in this context that polymethylmethacrylate (PMMA) is a biomaterial considered appropriate and widely used for making CDs [11]. Its unique characteristics make it one of the most important materials in the field of dental prosthesis. Despite this, the use of CDs may modify the oral microbiota, with an increase in the number of oral pathogens. Overall, most studies in this field have focused on Candida as the main cause of oral disorders [12–15], while a much smaller number of studies have investigated the occurrence of disorders due to periodontal pathogens [16, 17]. Among these latter, not all have used methodologies that provide sufficient evidence of a cause and effect relationship. Yasui et al. [16], analyzed the microbial composition of denture patients with previous rehabilitation, comparing these findings with those of patients with natural teeth. Given that the authors did not conduct any microbial examination prior to the use of dentures for the first time, their findings and conclusions do not support the hypothesis of increased periodontal pathogen loads due to the use of CDs.

Here, the present paper attempts to deal with this kind of inconsistency by presenting a pilot study aiming to add important insights to the existing research on periodontal pathogens in denture patients. In light of this, our study was designed to evaluate RCB loads in edentulous patients, before and after dentures’ insertion.

## Materials and methods

### Ethical approval

The research was approved by the Ethics Board of the University Dentistry Clinical Center of the Republic of Kosovo with protocol number 378/2019. Participation in the study was voluntary. All patients were informed about the purpose of the study and gave their written consent to participate in it.

### Study participants

The present pilot study was carried out from September 2021 to February 2022 at the Department of Prosthodontics at the University Dental Clinical Center in Pristina, Republic of Kosovo. A total of thirty patients who were treated with CDs participated in the study. Inclusion criteria were as follows: (1) fully edentulous dental arches; (2) healthy oral mucosa (without inflammatory changes or any oral pathology). Exclusion criteria were: (1) high alveolar bone loss; (2) use of immunosuppressants; (3) brain disorders in old age; (4) previous use of CDs; (5) antibiotic therapy in the last 3 months.

CDs were made with PMMA material by using the heat curing technique. Patients were instructed to remove the dentures for 8 h a day (during the night sleep), as well as to clean them.

### Microbiological examination

**Fig. 1:**
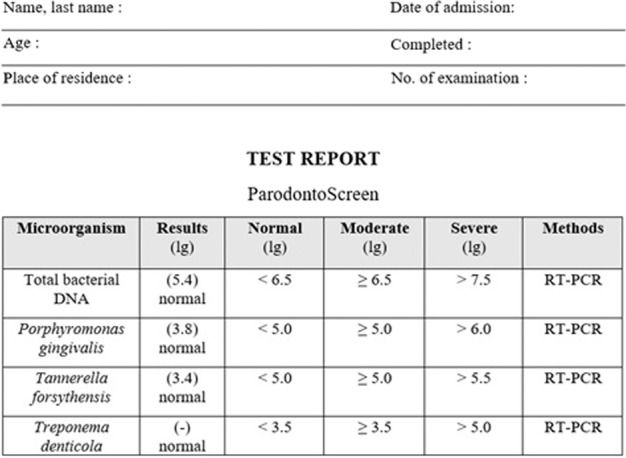
ParodontoScreen test report form. Interpretation of RT-PCR results for each RCB microorganism based on the ParodontoScreen test.

Microbiological data were analyzed before and 3 months after CDs insertion. The samples were taken from the dorsum of the tongue with a swab, which was rubbed several times for 45 s on a surface (dorsum of the tongue) of 1 cm^2^. The samples were obtained in the morning before eating. Each sample was then placed in a sterile container containing 2 ml of 0.9% sodium chloride saline solution and later (within a short time) sent to the microbiology laboratory. Laboratory procedures included four steps: separation of the sample from the swab, isolation of bacterial deoxyribonucleic acid (DNA), detection of bacteria, and interpretation of results. The detection of the following bacteria: *Tannerella forsythia, Porphyromonas gingivalis, and Treponema denticola* was performed using the Parodontoscreen technology via real-time polymerase chain reaction (RT-PCR). This technology enables direct laboratory analysis to be carried out—thus a biological sample can be analyzed to determine the presence and quantity of DNA from opportunistic microflora. In this sense, it was also important to evaluate the sample intake control (SIC) to control the validity of the results through quantifying the human genomic DNA. In this case, if the SIC value is less than 2.5, the sample is considered to contain an insufficient amount of DNA for obtaining a reliable result. Data processing was performed using DTlite1 and DTprime2 REAL-TIME Thermal cyclers devices produced by “DNA-Technology”. The results were classified based on a test report previously prepared by the manufacturer (Fig. 1). Bacterial load values were determined for each periodontal pathogen using the lg unit (logarithm of the number of genomes per sample). More information about the Parodontoscreen technology is available in detail on the manufacturer’s website [18].

### Statistical analysis

Series with attributive variables (gender, education, diabetes, smoking, and prevalence of RCB) were analyzed as percentage (%), while numerical series (age, and bacterial load) were described using Descriptive Statistics (Mean, Standard Deviation (SD); ± 95.00% confidence interval (CI); Minimum; Maximum). The difference in bacterial load values before and 3 months after denture use was analyzed using Wilcoxon Matched Pairs Test (Z/p) and Sign Test (Z/p). The Fisher’s exact test (Monte Carlo two-sided *p* values) was performed to analyze the discrepancies between the attribution series. All *p* values < 0.05 were considered significant.

## Results

### Study population

Thirty edentulous patients were treated with conventional removable CDs in both jaws. The patient group included 17 (56.7%) men and 13 (43.3%) women. The distribution of other demographic parameters is shown in Table 1.

**Table 1:** Study population demographics.

	*n* = 30
Gender n (%)	
Male	17 (56.7%)
Female	13 (43.3%)
Age (years)	
Mean ± SD	65.33 ± 6.58
Range	57–82
Diabetes, n (%)	
Yes	16 (53.3%)
No	14 (46.7%)
Smokers, n (%)	
Yes	8 (26.7%)
No	22 (73.3 %)
Education, n (%)	
Primary	10 (33.3%)
Secondary	18 (60.0%)
High	2 (6.7%)

*SD* standard deviation.

### Microbiological examination

**Fig. 2:**
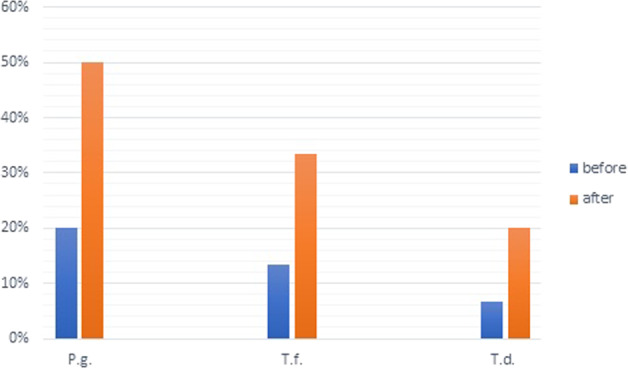
Distribution of positive samples of RCB before and 3 months after the CDs insertion. P.g. Porphyromonas gingivalis; T.f. Tannerella forsythia; T.d. Treponema denticola.

The frequency of periodontal pathogens before and after dentures’ insertion is shown in Fig. 2. The frequency of RCB in our samples before the CDs insertion was as follows: *Porphyromonas gingivalis* was identified in 6 (20%) samples, *Tannerella forsythia* in 4 (13.3%), and *Treponema denticola* in 2 (6.6%). After 3 months of the CDs insertion, we observed that the frequency of RCB increased considerably, with *P. gingivalis* being detected in 15 (50%) of the samples, *T. forsythia* in 10 (33.3%), and *T. denticola* in 6 (20%).

The bacterial load values for all RCB are described in Table 2. The bacterial load value for *P. gingivalis* ranged from 0.40 to ±0.90 Lg; ±95.00% CI: 0.06–0.73; with a minimum value of 0 Lg and a maximum value of 3.70 Lg. After 3 months of the CDs insertion, the bacterial load value for *P. gingivalis* varied in the range of 1.29 ± 1.64 Lg; ±95.00% CI: 0.67–1.90; while the minimum value found was 0 Lg and the maximum value was 5.00 Lg. A significant increase was observed for the load of all bacteria after 3 months of the CDs insertion (*p* < 0.05) (Table 2).

**Table 2:** Bacterial load values of periodontal pathogens before and 3 months after of treatment with CDs

(*n* = *30*)				
	Before	After	Z	*P*
*Porphyromonas gingivalis*				
Mean ± SD	0.40 ± 0.90	1.29 ± 1.64	3.41	0.0007
Range (lg)	0–3.7	0–5.0		
*Tannerella forsythia*				
Mean ± SD	0.36 ± 0.94	0.87 ± 1.45	2.80	0.005
Range (lg)	0–3.1	0–4.4		
*Treponema denticola*				
Mean ± SD	0.11 ± 0.41	0.33 ± 0.75	2.20	0.03
Range (lg)	0–1.8	0–2.8		

*lg* logarithm of the number of genomes, *SD* standard deviation, *Z and P* statistical variables.

The results presented in Table 3 show the RCB prevalence before and after CDs insertion. A normal bacterial prevalence range (<5.0 Lg) was found for *P. gingivalis* before the CDs insertion in the samples from all patients (100.0%). On the other hand, a moderate bacterial prevalence range (>5.0 Lg) for *P. gingivalis* was found in 2 (6.7%) samples after 3 months of the CDs insertion, while in the other 28 (93.3%) a normal range (<5.0 Lg) (Table 3) was observed. The results of the Fisher’s exact test (Monte Carlo two-sided *p* values), showed constant values.

**Table 3:** Prevalence of periodontal pathogens before and after 3 months of treatment with CDs.

	Bacteria prevalence				
	Before		After (*n* = 30)		
			Severe	Moderate	Normal
*Porphyromonas* *gingivalis*	Normal	Count	0	2	28
		%	0.00%	6.70%	93.30%
*Tannerella* *forsythia*	Normal	Count	0	0	30
		%	0.00%	0.00%	100.00%
*Treponema* *denticola*	Normal	Count	0	0	30
		%	0.00%	0.00%	100.00%

## Discussion

In the present study, we analyzed samples from the dorsum of the tongue from edentulous patients by means of RT-PCR in two periods, before and 3 months after CDs insertion. The microorganisms analyzed included the following pathogens: *Tannerella forsythia, Porphyromonas gingivalis, and Treponema denticola*. The colonization by RCB in edentulous patients can be initially attributed to the morphology of the dorsum of the tongue, which is rich in papillae and fissures, providing a favorable environment for bacterial colonization. Corroborating our study, Velden et al. [19] found in their research that the dorsum of the tongue can be a source of infection caused by periodontal pathogens, which is not still a consensus. The results of the experimental study by Danser et al. [4] did not indicate the presence of *P. gingivalis* after full-mouth extraction. In contrast to that, our findings are in agreement with different previous researches including those by Sachdeo et al. [5], Fernandes et al. [6] and Cortelli et al. [7], which using DNA extraction and analysis methods detected the presence of periodontal pathogens in edentulous patients. These studies clearly show the importance of molecular genetic methods for the detection of periodontal pathogens, providing robust evidence that such pathogens are able to colonize the oral cavity even in edentulous patients.

We also found in the present study a significant increase in RCB bacterial load after 3 months of the CDs insertion. The greatest increases were found for *P. gingivalis*, followed by *T. forsythia*, that are two microorganisms that have a strong relationship with each other [20]. Both bacteria, along with *T. denticola*, have an important impact on host cells with negative implications for the immune system, which may represent a great risk especially for the elderly [21]. Similar results were also found by Andjekovic et al. [17] who analyzed the bacterial load in the residual alveolar ridges of edentulous patients using PCR. The authors observed a significant increase in the bacterial load of *Actinobacillus actinomycetemcomitans, Prevotella intermedia*, and *T. forsythia* after 6 months of dentures insertion. In this case, the greater increases found in bacterial load can be attributed to the longer follow-up time of this study compared to ours.

Studies by some other researchers also corroborate our findings. Nair et al. [22]. evaluated bacterial contamination in different periods of use of removable dentures and concluded that the time of use significantly increases bacterial contamination. In contrast, the study by SAL Abdul-Kareem et al. [23]. demonstrated that the total number of microorganisms may decrease after 1 month of CDs insertion in edentulous patients. These discrepant findings reinforce the need and importance of further research in order to determine new insights into the influence of time of use on denture contamination.

Another explanation for the increase in bacterial load observed in our study must be related to the degree of roughness of the polymethylmethacrylate material used in the making of CDs. This is because the presence of pores on the denture surface increases the possibility of bacterial colonization [24]. It is expected that CDs insertion will affect the salivary flow and thus the denture to be covered by a layer called ‘pellicle’, which through specific interactions facilitates the colonization of bacteria, making the denture a source of infection [25]. Another important factor that can impact bacterial loads is the materials used to make CDs. In the present study, CDs were made by using the conventional heat-cured polymer. Arutyunov et al. [26] investigated microbial adhesion to different denture-making techniques using PMMA and found that the microbial adhesion index for hot-cured polymers was considered average compared to other methods.

Although all patients participating in our study received instructions on appropriate use and hygiene of the prosthesis, the bacterial load level was found to be high after a short period. One of the possible approaches to prevent bacterial colonization is the use of antimicrobial materials. Brown et al. [27] in their research concluded that regular use of denture cleaning tablets has the best effect in reducing polymicrobial biofilms. Thus, immersion of removable dentures in cleaning solutions may be useful and encouraged to complete denture cleaning.

Furthermore, based on the patient’s age, health status, and decline immune system with age, the results of the present study suggest that more importance should be given to all factors associated with bacterial colonization of CDs, given that the spread of RCB into the bloodstream may represent an increased risk in the development of several systemic diseases [8, 28].

### Limitations, strengths and future directions

A limitation of the present study is the inclusion of smokers and diabetes patients, as well as the fact that the study was carried out during the coronavirus disease 2019 pandemic. All these conditions have been shown in previous research to have the ability to impact the colonization of microorganisms, which may have influenced the results of this study [29–31].

Moreover, the evaluation of the loads of other periodontal pathogens besides the RCB was not conducted, which does not allow extrapolating our results to the entire population of periodontal pathogens. Fungi, including *Candida albicans*, were also not evaluated, which limits our conclusions about the loads of these microorganisms in view of their interactions with periodontal pathogens within the context studied here. However, our findings showed important and robust evidence that denture treatment in edentulous patients is associated with significant changes in the levels of RCB. Finally, we express our opinion that the present work provides a new concept for the future of implant-prosthetic planning in edentulous patients, especially considering the importance of these bacteria in the development of peri-implantitis [3].

## Conclusion

The use of CDs in edentulous patients may cause a significant increase in RCB loads. In light of this, a continuous follow-up of oral hygiene in edentulous patients is needed, including especially the cleaning of dentures and tongue, given that both are important sources of periodontal pathogens. The role of cleanliness in this case is essential to prevent not only oral health problems, but also the occurrence of systemic disorders.
